# Inhibiting BDNF/TrkB.T1 receptor improves resiniferatoxin-induced postherpetic neuralgia through decreasing ASIC3 signaling in dorsal root ganglia

**DOI:** 10.1186/s12974-021-02148-5

**Published:** 2021-04-19

**Authors:** Xiang Wei, Lina Wang, Jie Hua, Xiao-hong Jin, Fuhai Ji, Ke Peng, Bin Zhou, Jianping Yang, Xiao-wen Meng

**Affiliations:** 1 Department of Anesthesiology and Pain Management, The First Affiliated Hospital of Soochow University, 188 Shizi Street, Suzhou, Jiangsu China; 2 Jiangsu Key Laboratory of Clinical Immunology, Soochow University, Suzhou, Jiangsu China; 3 Jiangsu Key Laboratory of Gastrointestinal tumor Immunology, The First Affiliated Hospital of Soochow University, Suzhou, Jiangsu China

**Keywords:** Postherpetic neuralgia, Resiniferatoxin, Brain-derived neurotrophic factor, Truncated tropomyosin receptor kinase B receptor, Acid-sensitive ion channel 3, Neuronal excitability

## Abstract

**Background:**

Postherpetic neuralgia (PHN) is a devastating complication after varicella-zoster virus infection. Brain-derived neurotrophic factor (BDNF) has been shown to participate in the pathogenesis of PHN. A truncated isoform of the tropomyosin receptor kinase B (TrkB) receptor TrkB.T1, as a high-affinity receptor of BDNF, is upregulated in multiple nervous system injuries, and such upregulation is associated with pain. Acid-sensitive ion channel 3 (ASIC3) is involved in chronic neuropathic pain, but its relation with BDNF/TrkB.T1 in the peripheral nervous system (PNS) during PHN is unclear. This study aimed to investigate whether BDNF/TrkB.T1 contributes to PHN through regulating ASIC3 signaling in dorsal root ganglia (DRGs).

**Methods:**

Resiniferatoxin (RTX) was used to induce rat PHN models. Mechanical allodynia was assessed by measuring the paw withdrawal thresholds (PWTs). Thermal hyperalgesia was determined by detecting the paw withdrawal latencies (PWLs). We evaluated the effects of TrkB.T1-ASIC3 signaling inhibition on the behavior, neuronal excitability, and inflammatory response during RTX-induced PHN. ASIC3 short hairpin RNA (shRNA) transfection was used to investigate the effect of exogenous BDNF on inflammatory response in cultured PC-12 cells.

**Results:**

RTX injection induced mechanical allodynia and upregulated the protein expression of BDNF, TrkB.T1, ASIC3, TRAF6, nNOS, and c-Fos, as well as increased neuronal excitability in DRGs. Inhibition of ASIC3 reversed the abovementioned effects of RTX, except for BDNF and TrkB.T1 protein expression. In addition, inhibition of TrkB.T1 blocked RTX-induced mechanical allodynia, activation of ASIC3 signaling, and hyperexcitability of neurons. RTX-induced BDNF upregulation was found in both neurons and satellite glia cells in DRGs. Furthermore, exogenous BDNF activated ASIC3 signaling, increased NO level, and enhanced IL-6, IL-1β, and TNF-α levels in PC-12 cells, which was blocked by shRNA-ASIC3 transfection.

**Conclusion:**

These findings demonstrate that inhibiting BDNF/TrkB.T1 reduced inflammation, decreased neuronal hyperexcitability, and improved mechanical allodynia through regulating the ASIC3 signaling pathway in DRGs, which may provide a novel therapeutic target for patients with PHN.

**Supplementary Information:**

The online version contains supplementary material available at 10.1186/s12974-021-02148-5.

## Introduction

Postherpetic neuralgia (PHN) is a chronic neuropathic pain syndrome induced by the reactivation of latent varicella-zoster virus infection. Its typical symptoms include continuous aching, burning, and allodynia [1]. PHN causes physical disability, emotional distress, and many other complications, which seriously debilitate the quality of life [2]. Current treatments for PHN include medical (systemic tricyclic antidepressants, topical lidocaine and capsaicin, anticonvulsants and opioids) and interventional therapies (subcutaneous botulinum toxin injections, nerve blocks and neurostimulation )[3]. However, there is still a lack of effective clinical therapy and up to 50% of patients with PHN are refractory to management [4]. Thus, it is meaningful to develop novel efficacious therapeutic strategies for preventing PHN.

Brain-derived neurotrophic factor (BDNF) as a member of the neurotrophin family is widely expressed in mammalian adult brain. And it plays a crucial role in the development of the central nervous system (CNS), influencing neuronal maintenance, neuronal survival, synaptic plasticity, and neurotransmitter regulation [5]. Thus, BDNF has become the most widely studied neurotrophin because of the roles in brain homeostasis, long-term potentiation, learning and memory, health, and multiple CNS disease [6–8]. Besides that, BDNF is also constitutively synthesized by neurons of dorsal root ganglion (DRG) in the peripheral nervous system (PNS) and anterogradely transported from the cell body to the terminals in the spinal cord [9]. Notably, BDNF contributes to the sensitization of pain-related pathways in primary sensory neurons, making it a potentially novel therapeutic target [9–11]. Emerging evidence indicated that the synthesis and release of BDNF in DRGs were significantly increased in the process of inflammatory pain, cancer pain and neuropathic pain [12–14]. It is well known that BDNF mediates its effects via two distinct classes of receptors: the endogenous high-affinity tropomyosin receptor kinase B (TrkB) and the low-affinity p75 neurotrophin receptor (p75NTR). BDNF/TrkB-mediated intracellular signals are involved in many neuronal aspects, including neuronal survival, synaptic plasticity, and neurogenesis-associated cognition in CNS [6]. Moreover, TrkB is also abundantly expressed in the primary sensory neurons [15, 16]. By acting through this receptor, BDNF participates in nociceptive pain-related signaling of primary sensory neurons [11, 15]. It is of interest to note that differential splicing during the transcription of the TrkB receptor gene could give rise to three readily identified isoforms, including the full-length isoform (TrkB.FL) and two truncated isoforms (TrkB.T1 and TrkB.T2) [17]. Among these isoforms, the most commonly expressed forms are the catalytically active TrkB.FL and a truncated receptor TrkB.T1. TrkB.T1 has been increasingly studied in various experimental models of neuropathic pain [17, 18]. Nevertheless, the detailed molecular mechanism that elicits signaling downstream of BDNF/TrkB.T1 in the development of PHN remains elusive.

Acid-sensitive ion channels (ASICs) are cation-selective channels that are widely expressed in peripheral and central nervous systems in mammals [19]. Some studies demonstrated that the ASIC1, ASIC2 and ASIC3 channel subtypes are mainly expressed in neurons using in situ hybridization experiments [20–22]. Among these subtypes, ASIC3 is the most abundant one in neurons of DRGs , as well as the most sensitive to extracellular acidification [23]. Sluka et al. [24] found ASIC3 knockout mice failed to develop chronic muscle pain in a mouse fibromyalgia model induced by repeated acid injections. Pro-inflammatory mediators, specifically nerve growth factor, interleukin-1, serotonin, and bradykinin, reportedly led to hyperexcitability of primary cultured DRG neurons through increasing ASIC3 expression [25]. Besides, BDNF from extracellular significantly increased excitability of cultured primary hippocampal neurons [26]. However, the exact regulation mechanism between BDNF/TrkB.T1 and ASIC3 channels in the primary sensory neurons during PHN needs to be explored.

Based on the strong feasibility of resiniferatoxin (RTX) and its possible mimic of the PHN-like symptoms [27, 28], we used RTX to induce PHN in the present study. We hypothesize that RTX injection increased the synthesis and secretion of BDNF, and then the upregulated BDNF activated ASIC3 signaling through the TrkB.T1 receptor. Our present study helps to demonstrate the mechanism underlying the role of BDNF/TrkB.T1-ASIC3 signaling which may be used as a potential therapeutic target for the prevention and treatment of PHN.

## Materials and methods

### Animals

Adult healthy male Sprague-Dawley rats weighing 200 ± 20 g were provided by the experimental animal center of Soochow University (Animal license No. SYXK Jiang-su 2017-0043). Animals were kept under controlled temperature of 24–26 °C, relative humidity 40–60%, and 12-h light-dark cycle with food and water available ad libitum. Animal care and handling were approved by the Institutional Animal Care and Use Committee of Soochow University (Suzhou, Jiangsu, China). All experiments were performed in accordance with the Guide for the Care of Use of Laboratory Animals published by the US National Institute of Health (NIH Publication No. 85-23, revised in 1996).

### Establishment of a resiniferatoxin-induced rat PHN model

Rats in the RTX group were received a single intraperitoneal injection of RTX (200 μg/kg, LC Laboratories, Woburn, MA, USA) with isoflurane inhalational (2% isoflurane in a mixture of oxygen and air) anesthesia as previously described [28]. RTX was dissolved in a mixture of 10% Tween 80 (Sigma, St. Louis, MO, USA) and 10% ethanol in normal saline. The rats in the vehicle group received the same volume of the mixture solution.

### Intrathecal injection

For intrathecal injection, the intrathecal catheter (PE-10 tube, Smiths Medical, Minneapolis, MN, USA) was administered 5 days before the establishment of the RTX-induced PHN model according to a previously described method [29] and our earlier study [30]. Animals were anesthetized by intraperitoneal injection of sodium pentobarbital (50 mg/kg) and properly fixed on the operation table. First, one 1-cm skin incision was made along the right side of the spinous process, and the epidural needle was inserted into the subarachnoid space of the spinal cord between the L4 and L5 spinous processes. Once the needle arrived at the location, an apparent resistance could be felt. Next, the spinal needle was inserted into the lumen of the epidural needle to make a small hole gently in the ligamentum flavum and dura mater with the loss of resistance. In addition, a reflexive flick of the tail was observed. Then, the polyethylene catheters were inserted into the side hole after withdrawing the spinal needle. Last, the catheter was firmly fixed to the spinous process and the muscles attached to the spinous process using silk suture. In addition, another 2-cm skin incision was made at the posterior midline of the neck. A 10-cm-long subcutaneous tunnel was made to induce the catheter to the back skin of the neck. Finally, the wound was closed in layers. All rats were returned to the animal facility and cared for by trained experimenters.

### Cell culture and BDNF treatment

Rat pheochromocytoma PC-12 cells were purchased from Stem Cell Bank, Chinese Academy of Sciences (Shanghai, China). Cells were cultured in Roswell Park Memorial Institute (RPMI)-1640 medium (Invitrogen, Carlsbad, CA, USA) containing 10% (v/v) heat-inactivated fetal bovine serum (Gibco, Life Technologies, Carlsbad, CA, USA), penicillin (100U/mL) and streptomycin (100μg/mL) in an incubator (Thermo Fisher Scientific, Waltham, MA, USA) at 37 °C with 5% CO_2_. Cells were treated with BDNF (2, 20, and 200 ng/mL, PeproTech, Cranbury, NJ, USA) for 20 min.

### ASIC3-shRNA transfection

Part I: For ASIC3 knockdown in PC-12 cells, cells were transfected with the short hairpin RNAs (shRNAs, Genechem, Shanghai, China) targeting ASIC3 (NM_173135). And cells were randomly divided into 4 groups: NC group (negative control transfection for 24h), shRNA1 group, shRNA2 group, and shRNA3 group (shRNAs-ASIC3 transfection for 24h, respectively). The PC-12 cells were seeded in six-well plates at a concentration of 4 × 10^5^ cells per well and cultured at 37 °C for 24 h to reach 70–80% confluent. Then, cells were washed with phosphate-buffered saline (PBS) three times and incubated with jetOPTIMUS® transfection complex (2μg DNA and 2 μL reagent in 200 μL jetOPTIMUS® buffer, Polyplus-transfection®SA, Strasbourg, Bas-Rhin, France) following the manufacturer’s protocol. Four hours later, the medium was changed to an antibiotic-free culture medium. After transfection for 24 hours, we observed transfection efficiency by using a fluorescent microscope. ASIC3 shRNA knockdown efficiency was confirmed by RT-PCR and western blotting analysis. The shRNA oligo sequences are shown in Table 1.

**Table 1:** Sequences of shRNA oligonucleotides targeting ASIC3

Name	Direction	oligonucleotides sequences 5′-3′
shRNA1	Sense	CCGGCAGCTGTGACTCTGTGTAATACTCGAGTATTACACAGAGTCACAGCTGTTTTTG
	Antisense	AATTCAAAAACAGCTGTGACTCTGTGTAATACTCGAGTATTACACAGAGTCACAGCTG
shRNA2	Sense	CCGGTGGCAACGGACTGGAGATTATCTCGAGATAATCTCCAGTCCGTTGCCATTTTTG
	Antisense	AATTCAAAAATGGCAACGGACTGGAGATTATCTCGAGATAATCTCCAGTCCGTTGCCA
shRNA3	Sense	CCGGTGGCTGTCGAATGATGCATATCTCGAGATATGCATCATTCGACAGCCATTTTTG
	Antisense	AATTCAAAAATGGCTGTCGAATGATGCATATCTCGAGATATGCATCATTCGACAGCCA

Part II: To investigate the effects of ASIC3 knock-down by shRNA on PC-12 cells during treatment with BDNF, cells were randomly divided into 4 groups: control group, BDNF group (cells were incubated with 20ng/ml BDNF for 20min), BDNF+shRNA-ASIC3 group (cells were transfected with shRNA for 24 h prior to BDNF treatment), and BDNF+NC (negative control) group.

### Behavioral tests

Mechanical allodynia was assessed using von Frey filaments (Stoelting Company, Wood Dale, IL, USA) as previously described [29]. Rat hindpaw was stimulated with a series of von Frey fiber (1, 1.4, 2, 4, 6, 8, 10, and 15 g). The paw withdrawal thresholds (PWTs) were defined as the lowest force of the filaments that produced at least three withdrawal responses in five tests. Thermal hyperalgesia was assessed by measuring the paw withdrawal latencies (PWLs) in response to a radiant heat source (Ugo Basile, Gemonio, Italy). The investigators were blinded to group allocation during data collection and analysis.

### Immunofluorescence

Animals were anesthetized with sodium pentobarbital (50 mg/kg, i.p.) and transcardially perfused with normal saline followed by 4% paraformaldehyde. The L4-L6 segments of DRGs were removed and post-fixed at 4 °C for 12 h, and then were dehydrated at 4 °C overnight with 30% sucrose solution in phosphate-buffered saline (PBS). DRG slices of 10 μm thick were obtained by using a cryostat. For double immunofluorescence, the slices were incubated with primary antibodies: rabbit anti-TrkB.T1 (1:200; Abcam, ab18987, Cambridge, MA, USA), rabbit anti-ASIC3 (1:100, Abcam, ab190638), rabbit anti-BDNF (1:200; Abcam, ab108319), mouse anti-neuronal nuclear protein (NeuN, 1:200, Abcam, ab104224), and mouse anti- Glial Fibrillary Acidic Protein (GFAP, 1:500, #3670, Cell Signaling Technology, USA). Images were captured under a fluorescence microscope (Nikon Corporation; Tokyo, Japan), and analyzed with the Image-Pro Plus software (Media Cybernetics, Silver Spring, MD, USA).

### Western blotting

The L4–L6 segments of DRG were rapidly dissected out and total protein was extracted by using the RIPA reagents (Beyotime, Shanghai, China). Then, the protein concentration was determined with a bicinchoninic acid protein assay kit (Beyotime, Shanghai, China). The tissue protein was separated by sodium dodecyl sulfate-polyacrylamide (SDS-PAGE) gel electrophoresis and transferred onto polyvinylidene fluoride (PVDF) membrane (Millipore Corp., Bedford, MA). The membrane was incubated with the primary antibodies overnight at 4 °C, then incubated with the horseradish peroxidase (HRP)-conjugated secondary antibodies for 2 h at room temperature. Finally, the densities of protein were normalized to β-actin as control. The primary antibodies were used as the following: rabbit anti-ASIC3 (1:500, Abcam, ab190638), rabbit anti-TrkB.T1(1:1000, Abcam, ab18987), rabbit anti-TrkB.FL (1:1000; Abcam, ab18987, Cambridge, MA, USA), rabbit anti-p-TrkB.FL T1 (1:1000; Abcam, ab229908, Cambridge, MA, USA), rabbit anti-tumor necrosis factor receptor-associated factor 6 (TRAF6, 1:1000, Abcam, ab33915), rabbit anti- neuronal nitric oxide synthase (nNOS, 1:1000, C12H1, Cell Signaling Technology, Beverly, MA, USA), rabbit anti-c-Fos (1:500, SC-52, Santa Cruz Technology, USA), rabbit anti-BDNF (1:500; Abcam, ab108319), and mouse anti-β-actin(1:1000, 2148, Cell Signaling Technology, Beverly, MA, USA).

### Quantitative real-time PCR

Total RNA from L4–L6 DRG segments or PC-12 cells were extracted by using the Trizol reagent (Invitrogen; Thermo Fisher Scientific, Inc. Waltham, MA, USA). Absorbances at 260 and 280 nm were measured for RNA quantification and purity control. According to the manufacturer’s instructions, the reverse transcription was performed using the cDNA Synthesis Kit (Applied Biological Materials, Richmond, BC, Canada). RNA samples were added to 5× All-in-one and DEPC water to form a 20 μl reaction system for reverse transcription to cDNA. Quantitative PCR was conducted with EvaGreen qPCR MasterMix (Applied Biological Materials, Richmond, BC, Canada) in 10μl reaction volumes on Roche Light Cycler R480 System (Roche, Bedford, MA, USA). And 10 μl reaction volume contained 1.5 μl of cDNA, 5 μl Eva Green, 1 μl of each pair of primers, and 1.5 μl DEPC water. The cycle parameters were as follows: pre-denaturation at 95 °C for 10 s, denaturation at 58 °C for 15 s, and annealing at 75 °C for 20 s for 40 cycles. The value obtained for the target gene expression was normalized to β-actin and analyzed by the relative gene expression 2^−ΔΔCT^ method. And all the experiments were repeated three times. The primers were provided by Shanghai Sangon Co., Ltd., and the sequences were:

ASIC3: Forward primer: 5′-CTGGCAACGGACTGGAGATTA-3′, Reverse primer: 5′-TGTAGTAGCGCACGGGTTGG-3′;

TrkB.T1: Forward primer: 5′-GGACCACGCCAACTGACATCG-3′, Reverse primer: 5′-ACCACCACAGCATAGACCGAGAG-3′;

β-actin: Forward primer: 5′-TCTATCCTGGCCTCACTGTC-3′, Reverse primer: 5′-AACGCAGCTCAGTAACAGTCC-3′;

Interleukin (IL)-1β: Forward primer: 5′-ATCTCACAGCAGCATCTCGACAAG-3′, Reverse primer: 5′-CACACTAGCAGGTCGTCATCATCC -3′;

IL-6: Forward primer: 5′-CCAGTTGCCTTCTTGGGACT-3′, Reverse primer: 5′-GGTCTGTTGTGGGTGGTATCC-3′;

Tumor necrosis factor (TNF) -α: Forward primer: 5′-GCATGATCCGAGATGTGGAACTGG-3′, Reverse primer: 5′-CGCCACGAGCAGGAATGAGAAG-3′.

β-actin: Forward primer: 5′-TCTATCCTGGCCTCACTGTC-3′, Reverse primer: 5′-AACGCAGCTCAGTAACAGTCC-3′.

### Acute isolation of DRG neurons and whole-cell patch-clamp recording

After animals were anesthetized, the L4–L6 DRGs of rats were quickly removed and transferred to an ice-cold oxygenated fresh dissection solution. DRGs were incubated with the dissection solution containing collagenase D (1.8–2.0 mg/ml, Roche; Indianapolis, IN, USA) and 0.25% trypsin (Sigma; St. Louis, MO, USA) for 1.5 h at 37 °C. At the end of digestion, the cells were allowed to adhere for 45 min on a glass coverslip, and then the adherent DRG cells were placed in a Petri dish and attached to a table of a reverse microscope. Small- and medium-sized DRG neurons were harvested and used in our study. The excitability of DRG neurons was measured by the whole-cell patch-clamp recording technique with an EPC10 patch-clamp amplifier (HEKA Electronics, Lambrecht, Germany). The data were obtained and analyzed by Fit Master from HEKA Electronics.

### Determination of nitric oxide levels

Nitric oxide release was quantified from the concentration of nitrite, a stable metabolite of NO. PC12 cells at the logarithmic growth phase were seeded in 6-well plates a density of approximately 1 × 10^6^ cells/well overnight. Following the different interventions, the NO level was assessed using the Nitric Oxide Assay kit (Abcam, Cambridge, MA, USA), with triplicates for each sample. The final absorbance values were measured at 540nm wavelength using a microplate reader (Molecular Devices, Sunnyvale, CA, USA). The NO level of each sample was calculated by a standard curve generated according to the manufacturer’s guidance.

### Statistical analysis

All data were expressed as mean ± standard error of the mean (SEM) and analyzed using the GraphPad Prism software (version 7.0, GraphPad, San Diego, CA, USA). Statistical significance was determined by using Student’s *t*-test, one-way analysis of variance (ANOVA) followed by Tukey’s post hoc test, or two-way repeated measures ANOVA followed by Bonferroni’s post hoc test, as appropriate. *P* < 0.05 was considered statistically significant.

## Results

### RTX injection increased ASIC3 and TrkB.T1 expression in DRGs

**Fig. 1:**
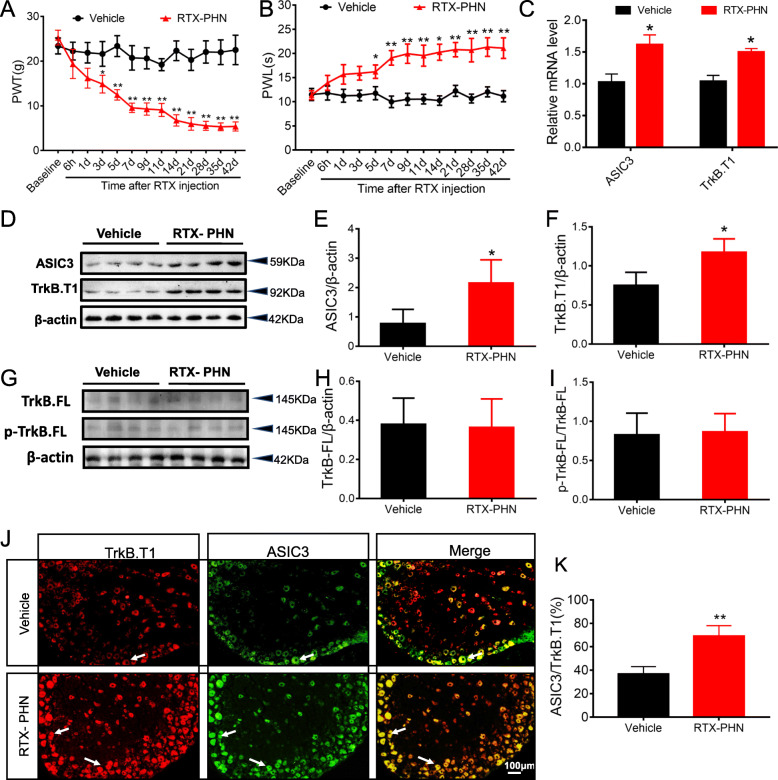
RTX injection increased ASIC3 and TrkB.T1 expression in DRGs. **a** Paw withdrawal thresholds (*n* = 10, *P < 0.05, **P < 0.01, two-way repeated measures ANOVA followed by Bonferroni’s post hoc test). **b** Paw withdrawal latency (*n* = 10, *P < 0.05, **P < 0.01, two-way repeated measures ANOVA followed by Bonferroni’s post hoc test). **c** Increased ASIC3 and TrkB.T1 mRNA expression after RTX injection (*n* = 4, *p < 0.05 vs. vehicle, Student’s *t*-test). **d**–**f** ASIC3 and TrkB.T1 protein expression in DRGs (*n* = 4, *p < 0.05 vs. vehicle, Student’s *t*-test). **g**–**i** TrkB.FL and p-TrkB.FL protein expression in DRGs (*n* = 4). **g** Merged double labeling of TrkB.T1 and ASIC3 (scale bar = 100 μm). **h** The percentage of ASIC3 labeled in TrkB.T1 positive cells (*n* = 5, *p < 0.05 vs. vehicle, Student’s *t*-test)

The effect of RTX administration on mechanical withdrawal threshold was examined using von Frey filaments. RTX significantly reduced the paw withdrawal thresholds (Baseline) from 3 days after injection (Fig. 1a). Meanwhile, the paw withdrawal latency to heat stimulus was obviously enhanced from 5 days after RTX treatment (Fig. 1b). In contrast, vehicle injection did not affect the rat paw withdrawal threshold or withdrawal latency. These results indicate the establishment of postherpetic neuralgia in rats [28, 31]. Then, DRGs were dissected out 14 days after RTX or vehicle injection. We selected day 14 to perform biochemical examinations for the following reasons. Firstly, the paw withdrawal threshold and withdrawal latency to heat stimulus at this time point become steady. Secondly, the ASIC3 mRNA level on day 14 was higher than the level on day 3 (supplementary Figure 1). Thirdly, we choose this time point not the day 42 because it also could minimize the animals’ duration of suffering from pain. RTX injection dramatically increased ASIC3 and TrkB.T1 mRNA expression (Fig. 1c) in DRGs. Western blot analysis showed that RTX injection also significantly enhanced the protein expression of ASIC3 and TrkB.T1 (Fig. 1d–f). Besides, we also detected the TrkB.FL and phosphorylated TrkB.FL(p-TrkB.FL) protein levels and found there was no significant change between the vehicle and RTX-PHN groups (Fig. 1g–i). Immunostaining was performed 14 days after RTX injection. As shown in Fig. 1j, TrkB.T1-positive cells (red) and ASIC3-positive cells (green) colocalized in the DRG neurons. Moreover, RTX injection significantly increased the number of positive neurons of TrkB.T1 and ASIC3 (Fig. 1k).

### Inhibition of ASIC3 attenuated mechanical allodynia and decreased TRAF6 signaling

**Fig. 2:**
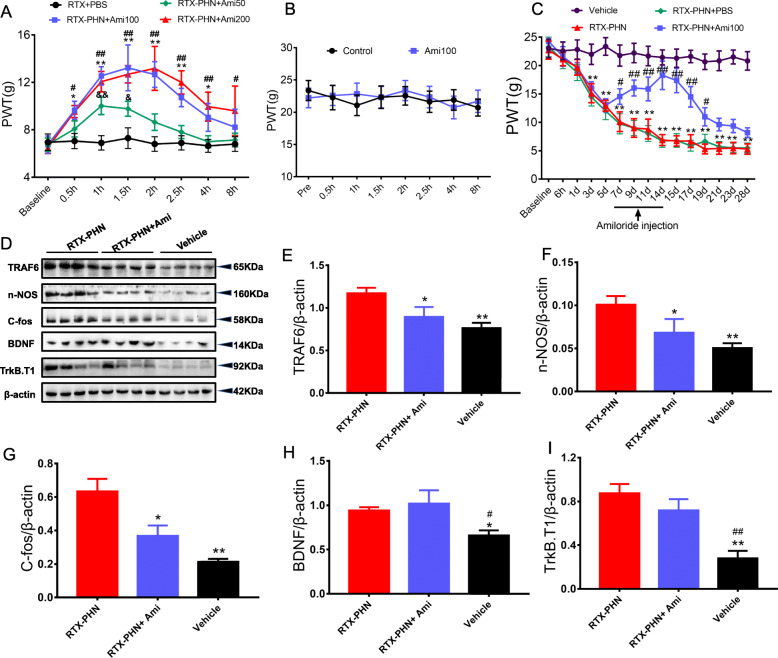
Inhibition of ASIC3 attenuated mechanical allodynia and decreased TRAF6 signaling. **a** Amiloride improved the paw withdrawal threshold (*n* = 10, ^&^*p* < 0.05, ^&&^*p* < 0.01 vs. RTX-PHN+PBS; *p < 0.05, **p < 0.01 vs. RTX-PHN+PBS; ^#^*p* <0.05, ^##^*p* <0.01 vs. RTX-PHN+PBS; two-way repeated measures ANOVA followed by Bonferroni’s post hoc test). **b** Amiloride at 100 μg had no significant effects on the mechanism threshold in healthy control rats(*n* = 10). **c** Amiloride (100 μg) administration with once per day for 7 consecutive days effectively blocked the decreased in paw withdrawal threshold (*n* = 10; **p < 0.01 vs. vehicle; ^#^*p* <0.05, ^##^*p* < 0.01 vs. RTX-PHN; two-way repeated measures ANOVA followed by Bonferroni’s post hoc test). **d**-**i** protein expression of TRAF6, nNOS, c-Fos, BDNF, and TrkB.T1 in DRG (*n* = 4, *p < 0.05, **p < 0.01 vs. RTX-PHN; ^#^*p* < 0.05, ^##^*p* < 0.01, vs. RTX-PHN+Ami; one-way ANOVA followed by Tukey’s post hoc test)

To determine whether ASIC3 is involved in RTX-induced postherpetic neuralgia, amiloride (Ami), a potent ASIC3 inhibitor, was intrathecally administrated at 14 days after RTX injection. Treatment with 50, 100, or 200 μg amiloride improved the paw withdrawal threshold in RTX-injected rats. The mechanism threshold was increased at 0.5 h and lasted for 8 h after Amiloride treatment in a dose-dependent manner (Fig. 2a). The use of 50 μg amiloride was less effective, and the effect of 200 μg amiloride was comparable with that of 100 μg. Meanwhile, 100 μg amiloride had no significant effects on the mechanism threshold in healthy control rats (Fig. 2b). Based on these results, 100 μg amiloride was administrated intrathecally from 7 days after RTX injection, once per day for 7 consecutive days. The ASIC3 inhibitor effectively blocked the decrease in the paw withdrawal threshold of RTX-injected rats (Fig. 2c). RTX injection also increased the protein expression of TRAF6 and nNOS, which was effectively reversed by consecutive amiloride administration. c-Fos, serving as a marker of neuronal activity, was detected. As shown in Fig. 2d, consecutive amiloride administration led to lower c-Fos protein level in DRGs compared with RTX injection (Fig. 2e–g). However, amiloride did not reverse the protein expression of BDNF and TrkB.T1 in DRGs induced by RTX injection (Fig. 2h, i).

### Inhibition of ASIC3 decreased hyperexcitability in DRGs neurons

**Fig. 3:**
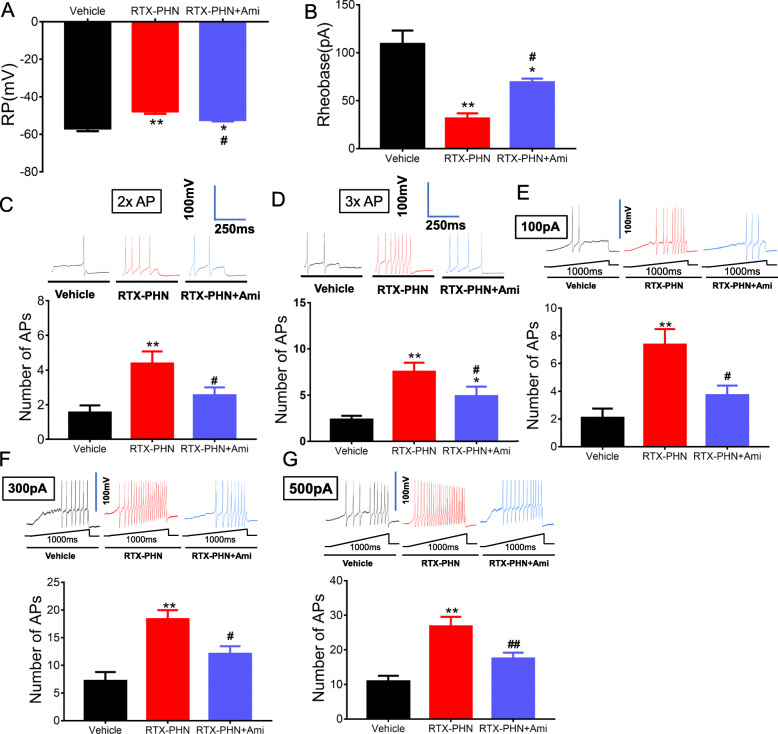
Inhibition of ASIC3 decreased hyperexcitability in DRGs neurons. **a** Amiloride reduced RP of DRG neurons. **b** Amiloride increased rheobase of DRG neurons. **c**, **d** Amiloride reversed numbers of APs in response to 2× and 3× rheobase current stimulation. **e**–**g** Amiloride reduced numbers of APs evoked by 100-, 300-, and 500-pA ramp current stimulation. *n* = 11, *p < 0.05, **p < 0.01, vs. vehicle group; ^#^*p* < 0.05, ^##^*p* < 0.01 vs. RTX-PHN; one-way ANOVA followed by Tukey’s post hoc test)

We next determined the intrinsic membrane properties including resting membrane potentials (RP), current threshold (rheobase), and pattern of firings in response to depolarizing current stimulation of DRG neurons. RTX injection significantly increased RP (Fig. 3a) and decreased rheobase (Fig. 3b), which was partly blocked by consecutively Amiloride administration. Besides, RTX injection obviously increased the number of action potentials (APs) in response to 2× rheobase current stimulation, as well as 3× rheobase current stimulation (Fig. 3 c and d), while treatment with amiloride reversed the increased response. In addition, the numbers of APs evoked by 100-, 300-, and 500-pA ramp current stimulation were determined. Amiloride significantly reduced the number of APs evoked by ramp current stimulation in DRG neurons of RTX-injected rats (Fig. 3e–g). Several additional membrane properties were also determined. Cell membrane capacitance (Cm), input resistance (Rin), AP threshold, AP duration, and overshoot were not significantly altered in DRG neurons from rats after vehicle, RTX, or amiloride injection (data not shown).

### Inhibition of TrkB.T1 attenuated mechanical allodynia and decreased ASIC3 signaling

**Fig. 4:**
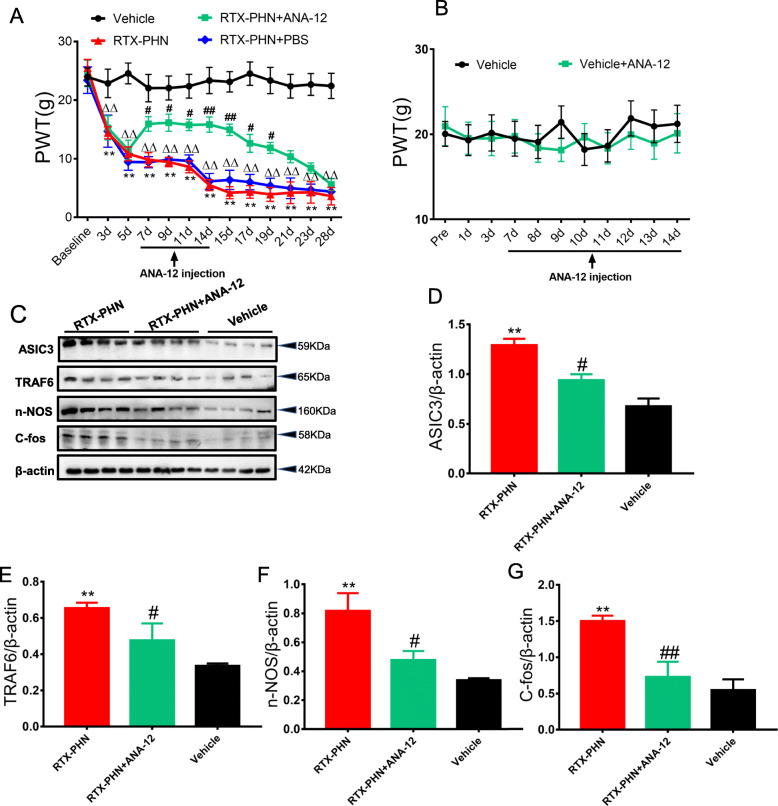
Inhibition of TrkB.T1 attenuated mechanical allodynia and decreased ASIC3 signaling. **a** ANA-12 increased paw withdrawal threshold (*n* = 10; **p < 0.01, ^∆∆^*p* < 0.01 vs. vehicle; ^#^*p* <0.05, ^##^*p* < 0.01 vs. RTX-PHN; two-way repeated measures ANOVA followed by Bonferroni’s post hoc test). **b** ANA-12 had no significant effects on the mechanism threshold in healthy control rats (*n* = 10). **c**–**g** Protein expression of ASIC3, TRAF6, nNOS and c-Fos in DRGs (*n* = 4, *p < 0.05, **p < 0.01 vs. vehicle, ^#^*p* <0.05, ^##^*p* <0.01 vs. RTX-PHN; one-way ANOVA followed by Tukey’s post hoc test)

ANA-12, a specific TrkB inhibitor, was intraperitoneally administrated from day 7 after RTX injection, once per day for 7 consecutive days. The ANA-12 effectively blocked the decreased paw withdrawal threshold induced by RTX injection (Fig. 4a). Besides, ANA-12 had no significant effects on the mechanism threshold in healthy control rats (Fig. 4b). ANA-12 also significantly inhibited the elevated protein expression of ASIC3, TRAF6, nNOS, and c-Fos induced by RTX injection (Fig. 4c–g).

### Inhibition of TrkB.T1 reduced hyperexcitability in DRGs neurons

**Fig. 5:**
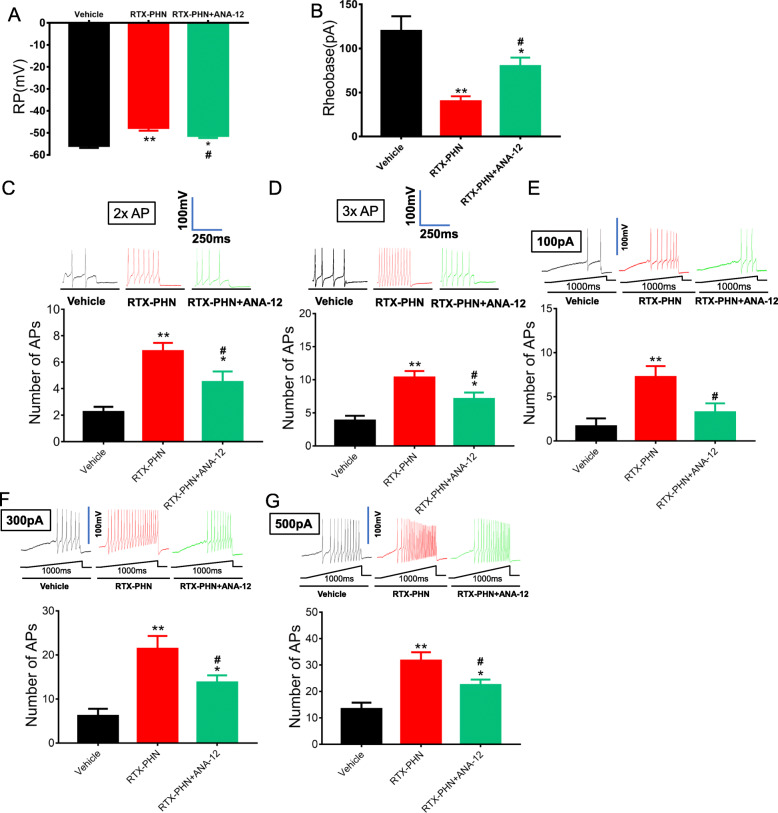
Inhibition of TrkB.T1 reduced hyperexcitability in DRGs neurons. **a** ANA-12 decreased RP of DRG neurons. **b** ANA-12 increased rheobase of DRG neurons. **c**, **d** ANA-12 reversed numbers of APs in response to 2× and 3× rheobase current stimulation. **e**–**g** ANA-12 reduced numbers of APs evoked by 100-, 300- and 500-pA ramp current stimulation. *n* = 12, *p < 0.05, **p < 0.01 vs. vehicle, ^#^*p* <0.05, ^##^*p* < 0.01 vs. RTX-PHN; one-way ANOVA followed by Tukey’s post hoc test

Next, ANA-12 significantly blocked the increased RP (Fig. 5a) and decreased rheobase (Fig. 5b) in DRG neurons induced by RTX injection. Besides, ANA-12 also decreased the number of APs in response to 2× rheobase current stimulation, as well as 3× rheobase current stimulation (Fig. 5 c and d), induced by RTX injection. In addition, ANA-12 significantly decreased the number of APs evoked by 100-, 300-, and 500-pA ramp current stimulation in DRG neurons of RTX-injected rats (Fig. 5e–g).

### TrkB.T1 receptor contributed to RTX-PHN through ASIC3 signaling

**Fig. 6:**
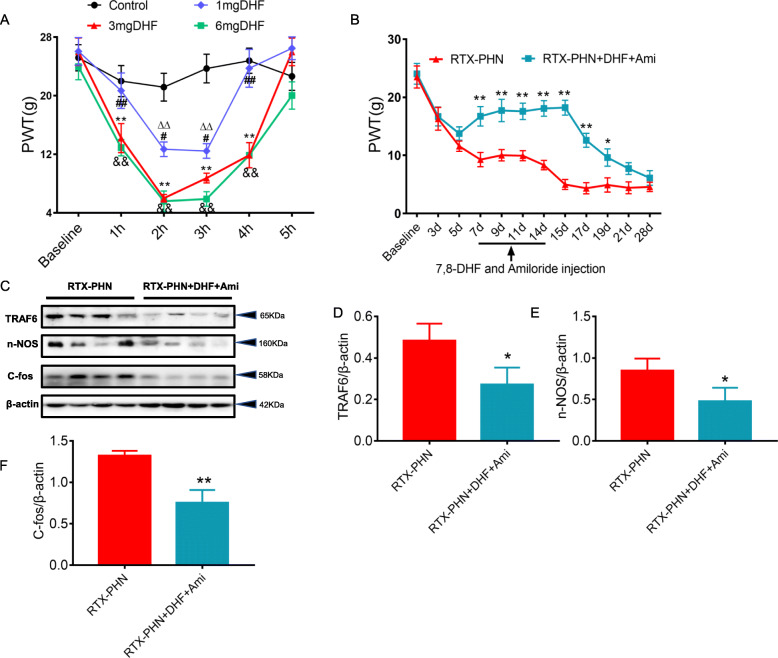
TrkB.T1 receptor contributed to RTX-PHN through ASIC3 signaling. **a** 7,8-DHF decreased the paw withdrawal threshold of healthy rats (*n* = 10; ^∆∆^*p* < 0.01, control vs. 1 mg DHF; **p < 0.01, control vs. 3mg DHF; ^&&^*p* < 0.01, control vs. 6 mg DHF; ^#^*p* <0.05, ^##^*p* <0.01, 1mg DHF vs. 3mg DHF; two-way repeated measures ANOVA followed by Bonferroni’s post hoc test). **b** 7,8-DHF and amiloride treatment reversed the decreased paw withdrawal threshold (*n* = 10; *p < 0.05, **p < 0.01 vs. RTX-PHN; two-way repeated measures ANOVA followed by Bonferroni’s post hoc test). **c**–**f** 7,8-DHF and Amiloride treatment inhibited protein expression of TRAF6, nNOS and c-Fos (*n* = 4, *p < 0.05, **p < 0.01 vs. RTX-PHN; Student’s *t*-test)

Next, 7,8-dihydroxyflavone (7,8-DHF), a potent small molecular TrkB agonist, was intrathecally administrated in healthy rats. 7,8-DHF (1, 3, or 6 mg/kg) significantly decreased the paw withdrawal threshold in healthy rats, and the threshold was decreased at 0.5 h and lasted for 4 h after 7,8-DHF treatment in a dose-dependent manner (Fig. 6a). Besides, 1 mg/kg 7,8-DHF was less effective, and the effect of 3 mg/kg was comparable with 6 mg/kg. Based on these results, 3 mg/kg 7,8-DHF, and 100 μg amiloride were administrated from day 7 after RTX injection, once per day for consecutive 7 days. The paw withdrawal threshold was significantly increased in RTX-PHN+DHF+Ami group compared with the RTX-PHN group (Fig. 6b). Consecutive administration of 3 mg/kg DHF and 100 μg amiloride also significantly inhibited the elevated protein expression of TRAF6, nNOS, and c-Fos induced by RTX injection (Fig. 6c–f).

### RTX injection increased BDNF expression in DRGs

**Fig. 7:**
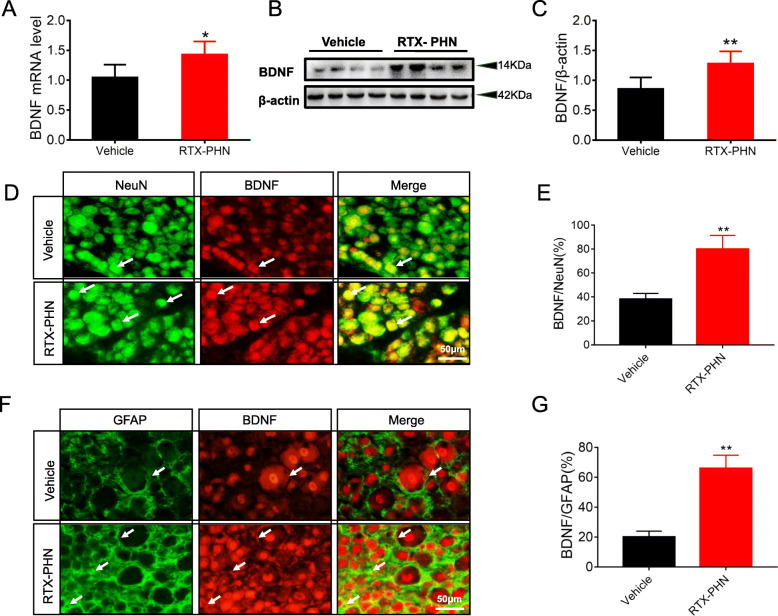
RTX injection increased BDNF expression in DRGs. **a** Increased BDNF mRNA expression after RTX injection (*n* = 4). **b**, **c** Increased BDNF protein expression in DRGs after RTX injection (*n* = 4). **d**, **e** Upregulated NeuN and BDNF protein co-expression after RTX injection. (**f**, **g**) Enhanced GFAP and BDNF protein co-expression after RTX injection (*n* = 5). *p < 0.05, **p < 0.01 vs. vehicle group, Student’s *t*-test. Scale bar = 50μm

DRGs were dissected out 14 days after RTX or vehicle injection. RTX injection dramatically increased BDNF mRNA expression (Fig. 7a) and enhanced BDNF protein expression in DRGs (Fig. 7 b and c). Next, double-labeling studies were performed 14 days after RTX injection. As shown in Fig. 7d, NeuN-positive cells (green) and BDNF-positive cells (red) colocalized in the DRG neurons (yellow), and the percentage of BDNF-positive cells in neurons were increased after RTX injection (Fig. 7e). Besides, we found that GFAP-positive cells (green) and BDNF-positive cells (red) also colocalized in the satellite glia cells; the percentage of BDNF-positive cells in satellite glia cells were increased after RTX injection (Fig. 7 f and g).

### Exogenous BDNF enhanced TrkB.T1-ASIC3 signaling in PC-12 cells

**Fig. 8:**
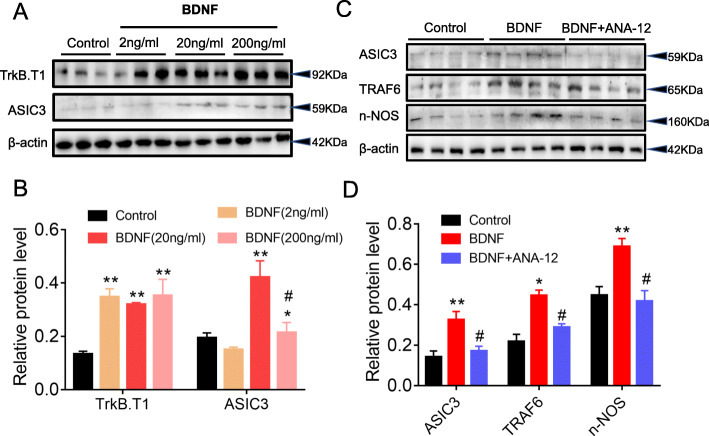
Exogenous BDNF enhanced TrkB.T1-ASIC3 signaling in PC-12 cells. **a**, **b** Exogenous BDNF increased TrkB.T1 and ASIC3 protein expression in PC-12 cells (*n* = 3, *p < 0.05, **p < 0.01 vs. control group; ^#^*p* < 0.05 vs. BDNF (20 ng/ml) group; one-way ANOVA followed by Tukey’s post hoc test). **c**, **d** ANA-12 decreased protein expressions of ASIC3, TRAF6 and nNOS (*n* = 4, *p < 0.05, **p < 0.01 vs. control group; ^#^*p* < 0.05 vs. BDNF group; one-way ANOVA followed by Tukey’s post hoc test)

To investigate whether the TrkB.T1-ASIC3 signaling activation in DRGs was induced by BDNF secretion from glia or neurons, the cultured PC-12 cells were incubated with exogenous BDNF for 20 min. Treatment with BDNF (2, 20, or 200 ng/ml) significantly increased TrkB.T1 protein expression. Meanwhile, both 20 and 200 ng/ml BDNF significantly increased ASIC3 protein expression, but 2 ng/ml BDNF did not. In addition, the use of 20 ng/ml BDNF was more effective than 200 ng/ml BDNF (Fig. 8 a and b). Based on these results, 20 ng/ml BDNF was used in the following experiments. Besides, exogenous BDNF upregulated the protein expressions of ASIC3, TRAF6, and nNOS in PC-12 cells, while these above changes were reversed by ANA-12 treatment (Fig. 8 c and d).

### Knockdown of ASCI3 abolished TRAF6 signaling activation induced by exogenous BDNF treatment in PC-12 cells

**Fig. 9:**
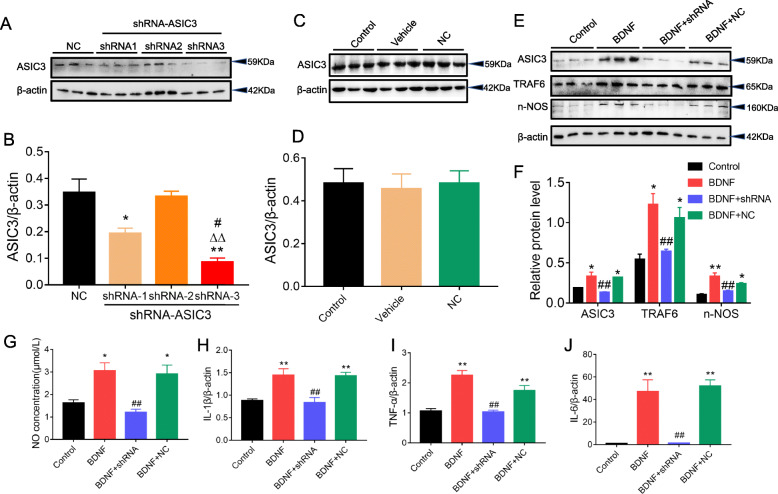
Knockdown of ASCI3 abolished TRAF6 signaling activation induced by exogenous BDNF treatment in PC-12 cells. **a**, **b** Transfection with shRNAs reduced ASIC3 protein levels (*n* = 3, *p < 0.05, **p < 0.01 vs. NC group; ^∆∆^*p* < 0.01 vs. shRNA2 group; ^#^*p* < 0.05 vs. shRNA1 group; one-way ANOVA followed by Tukey’s post hoc test). **c**, **d** ASIC3 protein levels in NC group was not changed compared with the control group (*n* = 3). **e**, **f** shRNA transfection inhibited the protein expressions of ASIC3, TRAF6 and nNOS induced by exogenous BDNF(*n* = 3). **g** Exogenous BDNF increased NO level, which was reversed by shRNA transfection(*n* = 5). **h**–**j** Exogenous BDNF upregulated mRNA expressions of IL-6, IL-1β and TNF-α, which was reversed by shRNA transfection(*n* = 5). *p < 0.05, **p < 0.01 vs. control group; ^#^*p* < 0.05, ^##^*p* < 0.01 vs. BDNF group; one-way ANOVA followed by Tukey’s post hoc test

Then, PC-12 cells were transfected with three different ASIC3 shRNAs (shRNA1, shRNA2, and shRNA3) for 24 h. Transfection with shRNAs significantly reduced ASIC3 protein levels compared with the NC groups, and western blot results showed that shRNA3 was the most efficient one to inhibit the protein expression of ASIC3 (Fig. 9 a and b), while the ASIC3 protein levels in the NC group was not changed compared with the control groups (Fig. 9 c and d). The shRNA3 was used in the following experiments, and we found that shRNA transfection significantly inhibited the increased protein expressions of ASIC3, TRAF6, and nNOS induced by exogenous BDNF (Fig. 9 e and f). Next, NO concentration was assessed using a nitric oxide assay kit. Exogenous BDNF also significantly increased the NO level, which was reversed by shRNA transfection (Fig. 9g). Besides, exogenous BDNF obviously upregulated mRNA expressions of IL-6, IL-1β, and TNF-α, which was reversed by knockdown of ASIC3 (Fig. 9h–j).

## Discussion

**Fig. 10:**
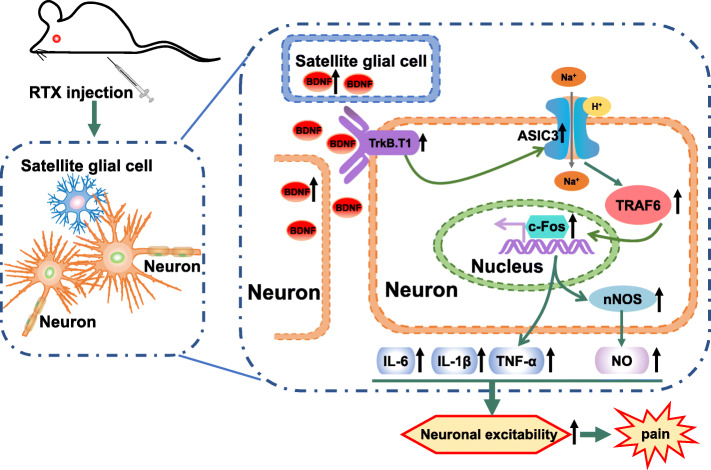
Schematic showing BDNF/TrkB.T1 contributes to RTX-induced postherpetic neuralgia through regulating ASIC3 signaling. RTX injection increased the synthesis and secretion of BDNF from activated satellite glial cells and neurons in DRGs. Then, BDNF activated ASIC3 signaling through increasing TrkB.T1 receptors in neurons, which led to elevated expression of c-FOS, TRAF6, and nNOS, as well as enhanced transcription of IL-6, IL-1β, TNF-α. Finally, the inflammatory response and NO production induced neuronal excitability and promoted the development and maintenance of pain

The BDNF-TrkB.T1 signaling was implicated in the pathological development and functional amelioration of various neuronal disorders. Moreover, BDNF overexpression worsens pathological conditions [32]. One study found that BDNF activity was augmented in cultured neurons incubated with sera from patients with PHN [33]. In addition, ASIC3 is an important protein in the pathogenesis of chronic pain diseases, making it a good candidate for a pain sensor [34, 35]. Based on the current findings, this study investigated the exact regulation mechanism between BDNF/TrkB.T1 and ASIC3 signaling in a rat RTX-PHN model. To the best of our knowledge, this is the first study to demonstrate that inhibiting BDNF/TrkB.T1 reduced inflammation factor and NO levels, reversed hyperexcitability of DRGs neurons, and improved mechanical allodynia through regulating ASIC3 signaling (Fig. 10).

Mamet et al. [25] found that chronic inflammatory pain elevates ASIC3 expression in DRGs neurons, and ASIC3 blocker produced beneficial effects on the suppression of hyperalgesia in mice [36] and rats [37] and acid-induced pain in human subjects [38]. In addition, one study demonstrated that hyperalgesia only occurs in wild-type mice but not in ASIC3-knockout ones with the mice model of joint inflammation [39]. These results were consistent with our current findings that ASIC3 expression was significantly increased in DRGs neuron of RTX-induced PHN rat model, and hyperalgesia was improved with amiloride administration, implicating the importance of ASIC3 in RTX-induced pain sensation. Recent studies have shown that TRAF6 is closely related to neuropathic pain caused by the primary or secondary damage or dysfunction of the peripheral nervous system [40–42]. Consistent with the known response of TRAF6 activation in the immune system, our findings indicated that TNF-α, IL-1β, IL-6, and NO levels were significantly upregulated in PC-12 cells in response to exogenous BDNF and further revealed these increased changes were effectively blocked by knockdown of ASIC3. Our results also indicated that RTX enhanced neuronal excitability and preventing ASIC3 activation reversed the enhanced neural hyperexcitability of DRG neurons. The possible explanation for these findings is that ASIC3 depolarizes the neuronal membrane and leads to an excitation of the neuron through regulating TRAF6-nNOS signaling.

TrkB.T1 binds BDNF with high affinity, and some previous evidence reported that TrkB.T1 was implicated in the pathobiology of neuropathic pain and the expression was upregulated in multiple nervous system injury models [17, 18, 43]. Studies showed that the TrkB.T1 receptor was increased in the spinal dorsal horn during inflammatory pain [44] or neuropathic pain [18]. However, little is known about the function of the TrkB.T1 receptor in the primary sensory neurons of DRG. In our present study, we detected BDNF and TrkB.T1 protein levels were enhanced in the DRGs after RTX injection. Considering that TrkB.FL also contributes to the nervous system development [45, 46], we detected the TrkB.FL and p-TrkB.FL protein levels, and the results indicated TrkB.FL did not participate in the RTX-induced PHN. Besides, one recent study suggested that ASIC3 transcripts in DRGs were upregulated in spinal cord injury mice, and the increased expression levels were reduced by anti-BDNF treatment [47]. Based on our current results, ANA-12 attenuated mechanical allodynia, decreased ASIC3 signaling, and reduced hyperexcitability in DRGs neurons of RTX-injected rats, which further indicates that the BDNF/TrkB.T1 pathway contributes to RTX-induced PHN through regulating ASIC3 in the primary sensory neurons. Another previous study demonstrated that elevating the extracellular concentration of BDNF significantly increased neuronal excitability of cultured primary hippocampal neurons [26]. Thus, BDNF/TrkB.T1-regulated ASIC3 delivery may be an important mechanism for channel reactivation during neuropathic pain conditions, and an increased amount of ASIC3 channels may further increase neuronal excitability [48].

BDNF is normally expressed and synthesized in neurons, and it could release from DRGs to provide neuronal development or participate in pain perception and sensitization [49]. A previous study showed that BDNF released from sensory neurons did not significantly contribute to acute pain, but was necessary for the transition from acute to chronic inflammatory pain and some neuropathic pain states [9]. Our results found that BDNF expression was upregulated in the neurons of DRGs at 14 days after RTX injection, and we speculated that BDNF mainly contributed to the chronic pain processing in rat PHN models. Interestingly, we also found that BDNF expression was also increased in the activated satellite glial cells, suggesting more studies are still needed to verify the pronociceptive role of BDNF in neuropathic pain processes in the peripheral nervous system using the conditional knockout mice. Besides, the mature BDNF detection via western blot analysis may yield cross-detection of other mature neurotrophins because a mature BDNF peptide is almost entirely a conserved nerve growth factor (NGF) domain which is shared with other neurotrophins (e.g., NGF, neurotrophin-3, and neurotrophin-4) [50]. Thus, the cultured PC-12 cells were incubated with recombinant BDNF, and we found that the TrkB.T1-ASIC3 signaling activation in DRGs was indeed mediated by the mature BDNF.

This study has several limitations. First, as this study aimed to explore whether targeting TrkB.T1 could affect RTX-induced postherpetic neuralgia through regulating ASIC3 signaling, the interaction between ASIC3 and TRAF6 was not evaluated. Second, the current results suggest that ASIC3 contributed to RTX-induced postherpetic neuralgia through enhancing the protein level. However, we did not detect whether the channel function of ASIC3 was involved in the postherpetic neuralgia through increasing ASIC3 current. Last, BDNF is synthesized by a subpopulation of unmyelinated primary afferents located in the DRGs, and some studies reported that anterograde transport of BDNF was released to the central terminals in the spinal cord dorsal horn, then interacted with receptors in spinal neurons to facilitate excitatory neurotransmission, or modulate inhibitory signaling [51]. Thus, the role of BDNF signaling in the spinal dorsal horn also needs to be investigated in further studies.

## Conclusion

This present study revealed that inhibiting BDNF/TrkB.T1 reduced the RTX-induced mechanical allodynia, decreased inflammation factor levels, and reversed hyperexcitability of DRGs neurons through regulating ASIC3 signaling, which may provide a potential novel therapeutic target for the prevention and treatment of patients with PHN.

## Supplementary Information

**Additional file 1.**

## Data Availability

The data used to support the findings of this study are available from the corresponding authors upon request.

## Abbreviations

PHN: Postherpetic neuralgia
BDNF: Brain-derived neurotrophic factor
TrkB: Tropomyosin receptor kinase B
TrkB.FL: Full-length isoform of TrkB
p-TrkB.FL: phosphorylated TrkB.FL
TrkB.T1: Truncated isoform 1 of TrkB
TrkB.T2: Truncated isoform 2 of TrkB
ASIC: Acid-sensitive ion channel
PNS: Peripheral nervous system
CNS: Central nervous system
DRGs: Dorsal root ganglia
RTX: Resiniferatoxin
PWTs: Paw withdrawal thresholds
PWLs: Paw withdrawal latencies
shRNA: Short hairpin RNA
RPMI: Roswell Park Memorial Institute
NeuN: Neuronal nuclear protein
GFAP: Glial fibrillary acidic protein
SDS-PAGE: Sodium dodecyl sulfate-polyacrylamide
HRP: Horseradish peroxidase
nNOS: Neuronal nitric oxide synthase
NO: Nitric oxide
TRAF6: TNF receptor–associated factor 6
RP: Resting membrane potentials
Aps: Action potentials
Cm: Cell membrane capacitance
Rin: input resistance
7,8-DHF: 7,8-Dihydroxyflavone
Ami: Amiloride
i.p.: Intraperitoneal
NC: Negative control
NGF: Nerve growth factor
PBS: Phosphate-buffered saline
IL: Interleukin
TNF: Tumor necrosis factor

## References

[1] Nagel MA, Gilden D (2014). Neurological complications of varicella zoster virus reactivation. Curr Opin Neurol.

[2] Tseng HF, Lewin B, Hales CM, Sy LS, Harpaz R, Bialek S, Luo Y, Jacobsen SJ, Reddy K, Huang PY, Zhang J, Anand S, Bauer EM, Chang J, Tartof SY (2015). Zoster vaccine and the risk of postherpetic neuralgia in patients who developed herpes zoster despite having received the zoster vaccine. J Infect Dis.

[3] Attal N, Bouhassira D (2015). Pharmacotherapy of neuropathic pain: which drugs, which treatment algorithms?. Pain.

[4] Ngo AL, Urits I, Yilmaz M, Fortier L, Anya A, Oh JH, Berger AA, Kassem H, Sanchez MG, Kaye AD, Urman RD, Herron EW, Cornett EM, Viswanath O (2020). Postherpetic neuralgia: current evidence on the topical film-forming spray with bupivacaine hydrochloride and a review of available treatment strategies. Adv Ther.

[5] Lima Giacobbo B, Doorduin J, Klein HC, Dierckx R, Bromberg E, de Vries EFJ (2019). Brain-derived neurotrophic factor in brain disorders: focus on neuroinflammation. Mol Neurobiol.

[6] Notaras M, van den Buuse M (2019). Brain-derived neurotrophic factor (BDNF): novel insights into Regulation and Genetic Variation. Neuroscientist.

[7] Notaras M, Hill R, van den Buuse M (2015). The BDNF gene Val66Met polymorphism as a modifier of psychiatric disorder susceptibility: progress and controversy. Mol Psychiatry.

[8] Notaras M, van den Buuse M (2020). Neurobiology of BDNF in fear memory, sensitivity to stress, and stress-related disorders. Mol Psychiatry.

[9] Sikandar S, Minett MS, Millet Q, Santana-Varela S, Lau J, Wood JN, Zhao J (2018). Brain-derived neurotrophic factor derived from sensory neurons plays a critical role in chronic pain. Brain.

[10] Nijs J, Meeus M, Versijpt J, Moens M, Bos I, Knaepen K, Meeusen R (2015). Brain-derived neurotrophic factor as a driving force behind neuroplasticity in neuropathic and central sensitization pain: a new therapeutic target?. Expert Opin Ther Targets.

[11] Merighi A, Salio C, Ghirri A, Lossi L, Ferrini F, Betelli C, Bardoni R (2008). BDNF as a pain modulator. Prog Neurobiol.

[12] Thibault K, Lin WK, Rancillac A, Fan M, Snollaerts T, Sordoillet V, Hamon M, Smith GM, Lenkei Z, Pezet S (2014). BDNF-dependent plasticity induced by peripheral inflammation in the primary sensory and the cingulate cortex triggers cold allodynia and reveals a major role for endogenous BDNF as a tuner of the affective aspect of pain. J Neurosci.

[13] Kras JV, Weisshaar CL, Quindlen J, Winkelstein BA (2013). Brain-derived neurotrophic factor is upregulated in the cervical dorsal root ganglia and spinal cord and contributes to the maintenance of pain from facet joint injury in the rat. J Neurosci Res.

[14] Tomotsuka N, Kaku R, Obata N, Matsuoka Y, Kanzaki H, Taniguchi A, Muto N, Omiya H, Itano Y, Sato T, Ichikawa H, Mizobuchi S, Morimatsu H (2014). Up-regulation of brain-derived neurotrophic factor in the dorsal root ganglion of the rat bone cancer pain model. J Pain Res.

[15] Wang H, Wei Y, Pu Y, Jiang D, Jiang X, Zhang Y, et al. Brain-derived neurotrophic factor stimulation of T-type Ca(2+) channels in sensory neurons contributes to increased peripheral pain sensitivity. Sci Signal. 2019;12. 10.1126/scisignal.aaw2300. 10.1126/scisignal.aaw2300 31551295

[16] Qiao L, Vizzard MA (2002). Up-regulation of tyrosine kinase (Trka, Trkb) receptor expression and phosphorylation in lumbosacral dorsal root ganglia after chronic spinal cord (T8-T10) injury. J Comp Neurol.

[17] Cao T, Matyas JJ, Renn CL, Faden AI, Dorsey SG, Wu J. Function and Mechanisms of Truncated BDNF Receptor TrkB.T1 in Neuropathic Pain. Cells. 2020:9. 10.3390/cells9051194 PMC7290366 32403409

[18] Wu J, Renn CL, Faden AI, Dorsey SG (2013). TrkB.T1 contributes to neuropathic pain after spinal cord injury through regulation of cell cycle pathways. J Neurosci.

[19] Shafaat OS, Winkler JR, Gray HB, Dougherty DA (2016). Photoactivation of an Acid-sensitive ion channel associated with vision and pain. Chembiochem.

[20] Lingueglia E, de Weille JR, Bassilana F, Heurteaux C, Sakai H, Waldmann R, Lazdunski M (1997). A modulatory subunit of acid sensing ion channels in brain and dorsal root ganglion cells. J Biol Chem.

[21] Waldmann R, Bassilana F, de Weille J, Champigny G, Heurteaux C, Lazdunski M (1997). Molecular cloning of a non-inactivating proton-gated Na+ channel specific for sensory neurons. J Biol Chem.

[22] Chen CC, England S, Akopian AN, Wood JN (1998). A sensory neuron-specific, proton-gated ion channel. Proc Natl Acad Sci U S A.

[23] Lee CH, Chen CC (2018). Roles of ASICs in Nociception and Proprioception. Adv Exp Med Biol.

[24] Sluka KA, Price MP, Breese NM, Stucky CL, Wemmie JA, Welsh MJ (2003). Chronic hyperalgesia induced by repeated acid injections in muscle is abolished by the loss of ASIC3, but not ASIC1. Pain.

[25] Mamet J, Baron A, Lazdunski M, Voilley N (2002). Proinflammatory mediators, stimulators of sensory neuron excitability via the expression of acid-sensing ion channels. J Neurosci.

[26] Guo Y, Su ZJ, Chen YK, Chai Z (2017). Brain-derived neurotrophic factor/neurotrophin 3 regulate axon initial segment location and affect neuronal excitability in cultured hippocampal neurons. J Neurochem.

[27] Pan HL, Khan GM, Alloway KD, Chen SR (2003). Resiniferatoxin induces paradoxical changes in thermal and mechanical sensitivities in rats: mechanism of action. J Neurosci.

[28] Wu CH, Lv ZT, Zhao Y, Gao Y, Li JQ, Gao F, Meng XF, Tian B, Shi J, Pan HL, Li M: Electroacupuncture improves thermal and mechanical sensitivities in a rat model of postherpetic neuralgia. Mol Pain 2013, 9:18. 10.1186/1744-8069-9-18 PMC3626545 23551937

[29] Yang Y, Li H, Li TT, Luo H, Gu XY, Lu N, Ji RR, Zhang YQ (2015). Delayed activation of spinal microglia contributes to the maintenance of bone cancer pain in female Wistar rats via P2X7 receptor and IL-18. J Neurosci.

[30] Hou Y, Wang L, Gao J, Jin X, Ji F, Yang J (2016). A modified procedure for lumbar intrathecal catheterization in rats. Neurol Res.

[31] Hong B, Sun J, Zheng H, Le Q, Wang C, Bai K, et al. Effect of tetrodotoxin pellets in a rat model of postherpetic neuralgia. Mar Drugs. 2018;16(6). 10.3390/md16060195. 10.3390/md16060195 PMC6025269 29874779

[32] Ziemlinska E, Kugler S, Schachner M, Wewior I, Czarkowska-Bauch J, Skup M (2014). Overexpression of BDNF increases excitability of the lumbar spinal network and leads to robust early locomotor recovery in completely spinalized rats. PLoS One.

[33] Hama Y, Shiraki K, Yoshida Y, Maruyama A, Yasuda M, Tsuda M, Honda M, Takahashi M, Higuchi H, Takasaki I, Daikoku T, Tsumoto T (2010). Antibody to varicella-zoster virus immediate-early protein 62 augments allodynia in zoster via brain-derived neurotrophic factor. J Virol.

[34] Yuan FL, Chen FH, Lu WG, Li X (2010). Acid-sensing ion channels 3: a potential therapeutic target for pain treatment in arthritis. Mol Biol Rep.

[35] Yen LT, Hsieh CL, Hsu HC, Lin YW (2017). Targeting ASIC3 for relieving mice fibromyalgia pain: roles of electroacupuncture, opioid, and adenosine. Sci Rep.

[36] Martinez-Rojas VA, Barragan-Iglesias P, Rocha-Gonzalez HI, Murbartian J, Granados-Soto V (2014). Role of TRPV1 and ASIC3 in formalin-induced secondary allodynia and hyperalgesia. Pharmacol Rep.

[37] Jeong S, Lee SH, Kim YO, Yoon MH (2013). Antinociceptive effects of amiloride and benzamil in neuropathic pain model rats. J Korean Med Sci.

[38] Jones NG, Slater R, Cadiou H, McNaughton P, McMahon SB (2004). Acid-induced pain and its modulation in humans. J Neurosci.

[39] Ikeuchi M, Kolker SJ, Burnes LA, Walder RY, Sluka KA (2008). Role of ASIC3 in the primary and secondary hyperalgesia produced by joint inflammation in mice. Pain.

[40] Lu Y, Jiang BC, Cao DL, Zhang ZJ, Zhang X, Ji RR, Gao YJ (2014). TRAF6 upregulation in spinal astrocytes maintains neuropathic pain by integrating TNF-alpha and IL-1beta signaling. Pain.

[41] Lu Y, Cao DL, Jiang BC, Yang T, Gao YJ (2015). MicroRNA-146a-5p attenuates neuropathic pain via suppressing TRAF6 signaling in the spinal cord. Brain Behav Immun.

[42] Wang Z, Liu F, Wei M, Qiu Y, Ma C, Shen L, Huang Y (2018). Chronic constriction injury-induced microRNA-146a-5p alleviates neuropathic pain through suppression of IRAK1/TRAF6 signaling pathway. J Neuroinflammation.

[43] Matyas JJ, O'Driscoll CM, Yu L, Coll-Miro M, Daugherty S, Renn CL, Faden AI, Dorsey SG, Wu J (2017). Truncated TrkB.T1-mediated astrocyte dysfunction contributes to impaired motor function and neuropathic pain after spinal cord injury. J Neurosci.

[44] Renn CL, Leitch CC, Dorsey SG (2009). In vivo evidence that truncated trkB.T1 participates in nociception. Mol Pain.

[45] Notaras M, Du X, Gogos J, van den Buuse M, Hill RA (2017). The BDNF Val66Met polymorphism regulates glucocorticoid-induced corticohippocampal remodeling and behavioral despair. Transl Psychiatry.

[46] Sasi M, Vignoli B, Canossa M, Blum R (2017). Neurobiology of local and intercellular BDNF signaling. Pflugers Arch.

[47] Wada N, Shimizu T, Shimizu N, Kurobe M, de Groat WC, Tyagi P, Kakizaki H, Yoshimura N (2019). Therapeutic effects of inhibition of brain-derived neurotrophic factor on voiding dysfunction in mice with spinal cord injury. Am J Physiol Renal Physiol.

[48] Xu TL, Duan B (2009). Calcium-permeable acid-sensing ion channel in nociceptive plasticity: a new target for pain control. Prog Neurobiol.

[49] Ernsberger U (2009). Role of neurotrophin signalling in the differentiation of neurons from dorsal root ganglia and sympathetic ganglia. Cell Tissue Res.

[50] Wiesmann C, de Vos AM (2001). Nerve growth factor: structure and function. Cell Mol Life Sci.

[51] Cho HJ, Kim JK, Zhou XF, Rush RA (1997). Increased brain-derived neurotrophic factor immunoreactivity in rat dorsal root ganglia and spinal cord following peripheral inflammation. Brain Res.

