# Supplementary figures and images for: Inhibiting BDNF/TrkB.T1 receptor improves resiniferatoxin-induced postherpetic neuralgia through decreasing ASIC3 signaling in dorsal root ganglia
Source: J Neuroinflammation. 2021 Apr 19;18:96. doi: 10.1186/s12974-021-02148-5 (PMC8054387; doi:10.1186/s12974-021-02148-5)

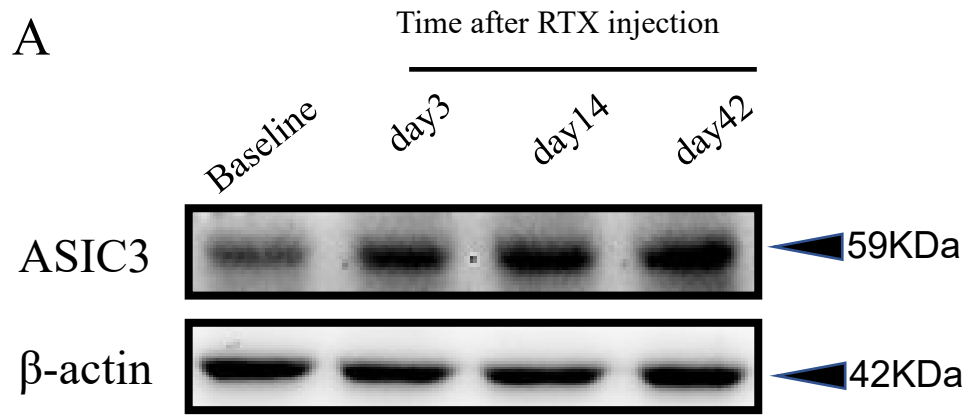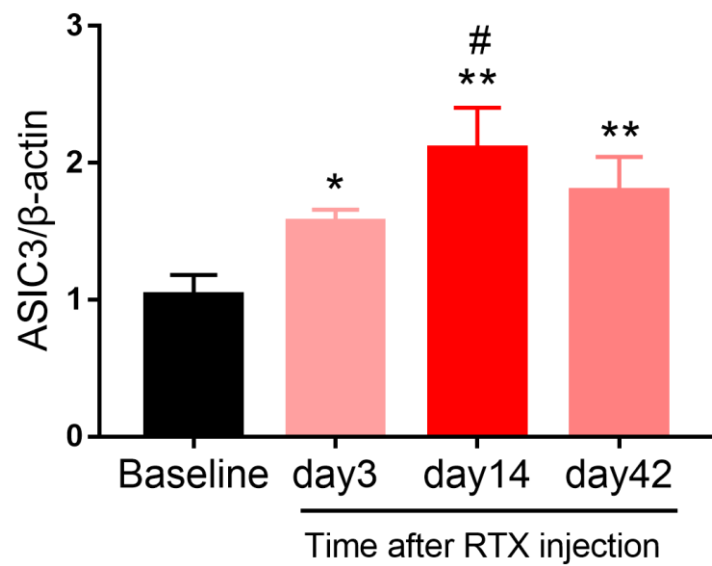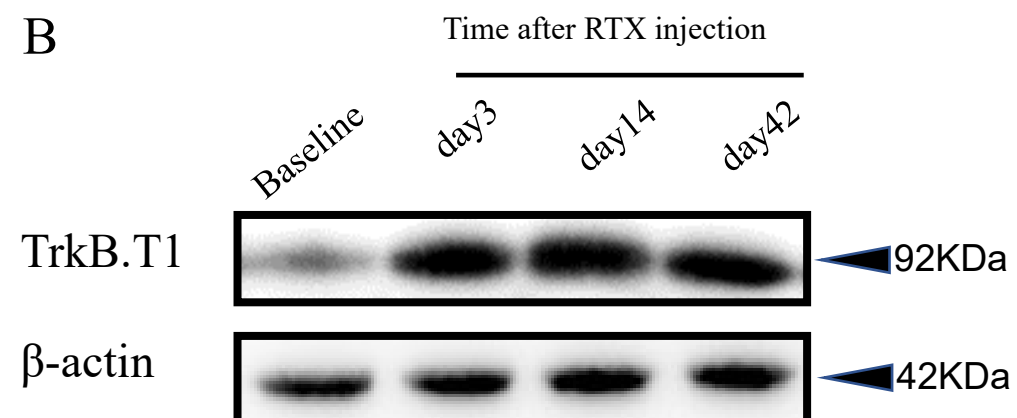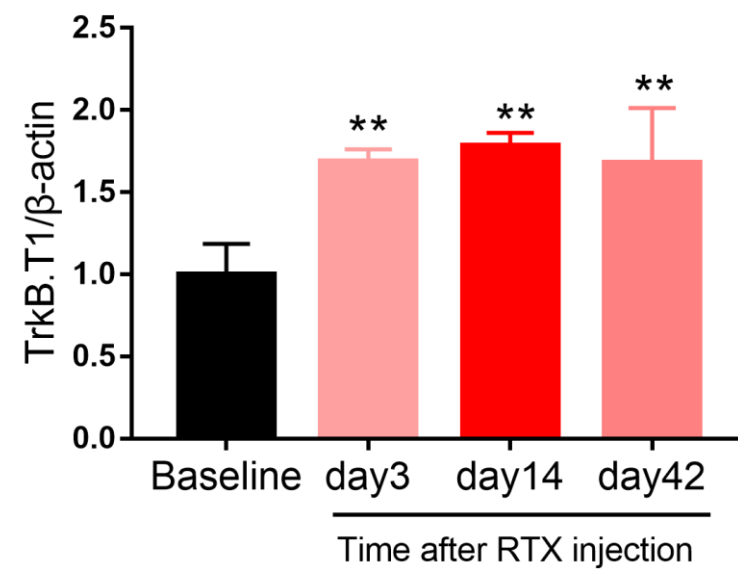

Supplement: Supplementary file 1 — Additional file 1. [file 12974_2021_2148_MOESM1_ESM.pdf]
